# The Illinois Training School for Nurses

**Published:** 1893-07-08

**Authors:** 


### THE INSTITUTIONAL WORKSHOP.

### WITHIN THE INSTITUTIONS.

### THE ILLINOIS TRAINING SCHOOL FOR NURSES.

(BY OUR SPECIAL COMMISSIONER.)

This institution has been established twelve years, and having inspected the hospitals of Chicago about the time it was instituted, we are in a position to assess with accuracy the value of the work done. Twelve years ago trained nurses were an unknown quantity in Chicago. Its chief hospital was the plaything of the ward politicians, and the lot of the poor man or woman in this city was therefore harder and worse than that of probably any city in the whole world. To-day, because the hand of the ward politician squeezes the life blood out of everything connected with the county hospital, except its nurses, the lot of the sick and poorest citizens of Chicago is neither happy nor just.

We have given this brief introduction because the Illinois Training School, through its devoted officers, has courageously taken up the cause of the sick poor, and at an expenditure of personal and loving service and self-denial on the part of both the nurses and their chiefs, much has been done to sweeten and cleanse the wards of the Cook County Hospital, and, in consequence, the patients, if under-fed, have for five years had the comfort of feeling there was help at hand and personal sympathy such as they never had experienced until this institution undertook the nursing. We would ask every Lady Superintendent and Matron to try and realise what it would be if she had to attempt to nurse patients in a hospital where the visiting staff seldom or never attended, and where the young resident internes were under no discipline, and where each worked for his own hand? This chaos means in practice that the nurse has sometimes for days to look after patients seriously ill who are neglected or ignored by the medical staff, and to see them suffer probably unnecessary and frequently preventable pain and risks to life, from this scandalous cause. In dealing with Cook County Hospital we shall give a full account of the conditions under which these devoted women have had to attempt to nurse Christ's poor at Chicago. Fancy what the strain must have been upon the Superintendent, Miss Edith A. Draper, and upon her assistants, especially when they were kept in daily remembrance of what could and ought to be done by the Medical Staff by their work at the Presbyterian Hospital. We can imagine nothing more wearing and exhausting than to have to labour day after day, and year after year, amid the sick and suffering under conditions which make one realise acutely what the sick in the hospital wards need, and to find that, despite every effort, those needs are neglected, and that every attempt to supply them usually proves futile because of the ward politician.

Lynching is a practice which has done much for the United States in many ways, and we make bold to say, after a careful inquiry and inspection of the Cook County Hospital, that if the poor citizens of Chicago were to rise and lynch the whole Board of Trustees of the Cook County Hospital they would take a step which the history of this institution seems to impel justice to bring about, unless radical reforms are enforced and the constitution altered forthwith.

Thanks to the devotion of the Superintendents and nurses of the Illinois Training School the wards of the Cook County Hospital are now kept clean and orderly. The patients present an appearance which shows how conscientious is the care which the nurses bestow upon them. The burden of the work thrown on the nurses must be immense. Not only are the wards cruelly overcrowded, but whenever the demand for admission arises, an unlimited number of beds is made up in every ward. This makes the work of nursing at this institution so herculean as to strike terror and excite despair in the minds of the ablest administrators. For five years Miss Draper has bravely faced the work and the present condition of the wards and patients at the Cook County Hospital eloquently testifies to the capacity she has for securing efficiency under the most distressing conditions. Her selection for the important post of Superintendent of the Great Victoria Hospital at Montreal, out of some 250 applications, is an honour to herself, and proves the capacity of the Board of Trustees who made this appointment.

#### THE NURSES AND THEIR WORK.

The Cook County Hospital contains 800 beds, of which 650 are occupied at the least busy season of the year. The Presbyterian Hospital contains 200 beds, which are constantly full. Nursing in both these institutions is undertaken by the Illinois Training School, 80 nurses being told off to work at the County, and 50 at the Presbyterian Hospital. The nurses are on duty nine hours out of the 24, although their day consists of 12 hours, the remaining three being occupied by meals, recreation, and rest. All nurses reside in the Nurses' Home, 304, Honoré Street. This home contains a large dining room, separate kitchen, and offices in the basement, with an adequate box-room and a room where the nurses change their things when they come off duty. On the ground floor there is a comfortable sitting-room which contains a library, that might be increased so far as the technical books are concerned, and the rooms of the Lady Superintendent and her assistants, with a few bed-rooms. The upper floors contain the bedrooms, lavatories, baths, &c. In Chicago the administration of this, the largest hospital, is at the lowest ebb, but the nursing exhibits a knowledge and efficiency which leaves little, if anything, to desire.

#### TRAINING: PRIVATE SERVICE AND DISTRICT NURSES.

Probationers receive two years' training at the Cook County and Presbyterian Hospitals. They are received in the school for one month on probation, with an option to the school authorities to extend the term of probation. Probationers are taken from twenty-three to thirty-five years of age, and during the month's trial examinations are conducted in reading, writing, simple arithmetic, and dictation. The Superintendent has full power to decide as to the probationer's fitness for the work, and the propriety of retaining or dismissing her at the end of the first month. She can also, with the approval of the committee, discharge the probationers at any time on account of misconduct or inefficiency. At the end of the first month, if accepted, the probationer is entered as a regular nurse on signing an agreement to remain two years and to obey the rules. The pupil nurses reside at the Home and serve for one year as assistants in the wards of the hospitals. But during the second year they are expected to perform any duty assigned them by the superintendent, either at the hospital or in attendance at private cases among the rich or poor. In addition to board and lodging, a free uniform is provided with the necessary class books, and each nurse receives with her diploma, after her final examination has been passed, 100 dols. (£20) in money. This grant is not regarded as payment for services rendered for which the training is considered an ample equivalent, but to enable young women without means to commence their career free from debt. The uniform is *de rigueur*, and consists of blue and white sersucker, white apron and cap, with linen collar and cuffs. The hours of duty are from half-past seven a.m. to half-past seven p.m., with an hour off for dinner and additional time for exercise or rest when possible. The nurses often have an afternoon during the week in addition to the half of each Sunday. Two weeks' vacation is allowed each year. No probationer is placed on duty until the expiration of the first three months of training. In case of sickness the nurses are treated gratuitously, but any time so occupied must be made up. The instruction includes the dressing of blisters, wounds. &c., the application of poultices, cups, &c., the management of helpless patients, bed making, bathing, prevention of bed sores, lifting, bandaging splints, and the preparation, cooking, and serving of delicacies for the sick. Nurses are also instructed in the principles of ventilation, warming and cleanliness, the actual observations and reports to be made to the physicians, and the management of convalescents. Instruction is given by the resident or visiting physicians. Examinations take place at regular intervals. When a nurse has graduated she is at liberty to choose her own field of labour, whether in hospitals or private families, or district nursing among the poor. A diploma is issued to each nurse signed by a committee of the board of managers, after the final examination has been passed. Any nurse who attends private patients must bring back a certificate of conduct and efficiency from the family, or from the medical attendant. The charge for a nurse is 3 dols. (12 shillings) per diem, or 15 dols. (£3) per week, in addition to travelling and washing expenses. The report contains a list of all the nurses who have graduated at the school, a practice which we hope will be generally adopted by hospitals in this country. The school has opened a directory, where the nurses can register the rates which they will render nursing services for, such rates ranging from 20 dols. (£4) per week upwards. Altogether, we were most favourably impressed with this institution, which has bravely carried on its difficult work under conditions some of which were little calculated to promote either efficiency or progress.

